# Reconstruction of Arm Movement Directions from Human Motor Cortex Using fMRI

**DOI:** 10.3389/fnins.2017.00434

**Published:** 2017-07-27

**Authors:** Seungkyu Nam, Dae-Shik Kim

**Affiliations:** Brain Reverse Engineering and Imaging Lab, School of Electrical Engineering, Korea Advanced Institute of Science and Technology Daejeon, South Korea

**Keywords:** fMRI, reconstruction, classification, decoding, encoding, directional movement

## Abstract

Recent advances in functional magnetic resonance imaging (fMRI) have been used to reconstruct cognitive states based on brain activity evoked by sensory or cognitive stimuli. To date, such decoding paradigms were mostly used for visual modalities. On the other hand, reconstructing functional brain activity in motor areas was primarily achieved through more invasive electrophysiological techniques. Here, we investigated whether non-invasive fMRI responses from human motor cortex can also be used to predict individual arm movements. To this end, we conducted fMRI studies in which participants moved their arm from a center position to one of eight target directions. Our results suggest that arm movement directions can be distinguished from the multivoxel patterns of fMRI responses in motor cortex. Furthermore, compared to multivoxel pattern analysis, encoding models were able to also reconstruct unknown movement directions from the predicted brain activity. We conclude for our study that non-invasive fMRI signal can be utilized to predict directional motor movements in human motor cortex.

## Introduction

Recent fMRI studies have successfully discriminated visual object categories (Haxby et al., 2001; Cox and Savoy, 2003), hand gestures (Dinstein et al., 2008), and visual features such as orientation and motion direction (Kamitani and Tong, 2005, 2006) from patterns of activity across an array of voxels. Similar methods have been used to reconstruct visual stimuli such as images or movies by modeling the brain activity in each voxel evoked by the visual stimuli (Thirion et al., 2006; Miyawaki et al., 2008; Naselaris et al., 2009; Nishimoto et al., 2011). On the other hand, decoding brain activity in motor areas usually require more invasive techniques. For example, invasive electrophysiological techniques have demonstrated that neuronal activities in human primary motor cortex (M1) can be used to control an artificial devices (Hochberg et al., 2006, 2012; Truccolo et al., 2008; Collinger et al., 2013). Such invasive techniques have been found to be more precise and intuitive when used to control an external effector using neuronal signals related to arm movements. Nonetheless, these methods inevitably involved considerable risks associated with surgical procedures and potential inflammations. Therefore, we used functional magnetic resonance imaging (fMRI) to measure brain signals non-invasively and investigated whether the recent decoding methods were applicable to motor areas.

Neurons in the macaque M1 are known to be broadly tuned to directional arm movements (Georgopoulos et al., 1982). This type of directional tuning is known as a basic functional property of neuronal activity in M1.Previous studies also demonstrated that human M1 neurons are sensitive to the movement direction based on electrophysiological signals (Hochberg et al., 2006; Truccolo et al., 2008). Furthermore, fMRI responses in human M1 suggested sensitivity to movement directions although each voxel contains a large number of neurons, where each of them has different selectivities (Eisenberg et al., 2010; Fabbri et al., 2010). Given that each voxel in motor cortex is directionally tuned, and despite the fact that the sensitivity of a voxel is weak, spatial patterns of fMRI response may be distinguishable for different movement directions. To this end, previous fMRI studies have used multivoxel pattern analysis (MVPA) based on linear classifier to discriminate cognitive states based on spatial patterns of fMRI responses (Mitchell et al., 2004; Haynes and Rees, 2006; Norman et al., 2006; Hansen, 2007; De Martino et al., 2008; Formisano et al., 2008; Haynes, 2009).

In this study, participants performed a center-out reaching task during fMRI scan. The participants moved their arm from a center position toward one of eight target positions repeatedly according to visual instructions. To investigate how head motions induced by repeated reaching movements have an effect on the identification of different movement directions, we also compared contralateral motor cortex with ipsilateral motor cortex which uninvolved in the center-out reaching task. The results demonstrated that reaching-out movements toward eight directions can be discriminated based on spatial patterns of fMRI responses in M1, although it was influenced by head motion artifacts. However, these methods were restricted to predicting sensory, cognitive, or motor information. In this model, spatial patterns of fMRI response are used to identify a specific task from a known stimulus set. To reconstruct an unknown stimulus, decoding methods using encoding model are applied. Encoding models use given stimuli to estimate corresponding brain activity in each voxel, and then are used to reconstruct unknown stimuli using the estimated fMRI responses. Here, we used the directional tuning properties in human M1 to estimate the brain activity evoked by directional motor movements in each voxel. The responses of each voxel were characterized as a linear combination of idealized directional tuning curves (Brouwer and Heeger, 2009). The identification and reconstruction of movement directions were performed using that linear encoding model. In the identification, the encoding model demonstrated similar performance compared to MVPA. To determine the feasibility of reconstructing all possible directional motor movements from a limited amount of pre-specified movement directions, we compared the reconstruction performance capabilities in the case when movement directions were used to estimate the encoding model with when they were not used.

## Materials and methods

### Participants

Eight healthy right-handed subjects (mean age, 24.25; range, 21–30 years) participated in the experiments, which consisted of functional scanning sessions for center-out reaching task along with a high-resolution anatomical scanning session. All subjects had normal visual acuity and no neurological or psychiatric history. They provided written informed consent regarding their participation. The experimental procedures were in compliance with the safety guidelines for MRI research and were approved by the Institutional Review Board for research involving human subjects at the Korea Advanced Institute of Science and Technology.

### MRI acquisition

The experiments were performed at 3T MR scanner (Siemens Magnetom Verio, Germany). The functional images were acquired with a T2^*^-weighted gradient recalled echo-planar imaging (EPI) sequence (TR, 1,000 ms; TE, 20 ms; flip angle, 90°; FOV: 64 × 64 mm; voxel size, 3 × 3 × 5.5 mm, number of slices, 21). T1-weighted magnetization-prepared rapid-acquisition gradient echo (MPRAGE) images were also acquired (TR, 1,800 ms; TE, 2.52 ms; FA, 9°; FOV, 256 × 256 mm; voxel size, 1 × 1 × 1 mm).

### Experimental design

**Figure 1:**
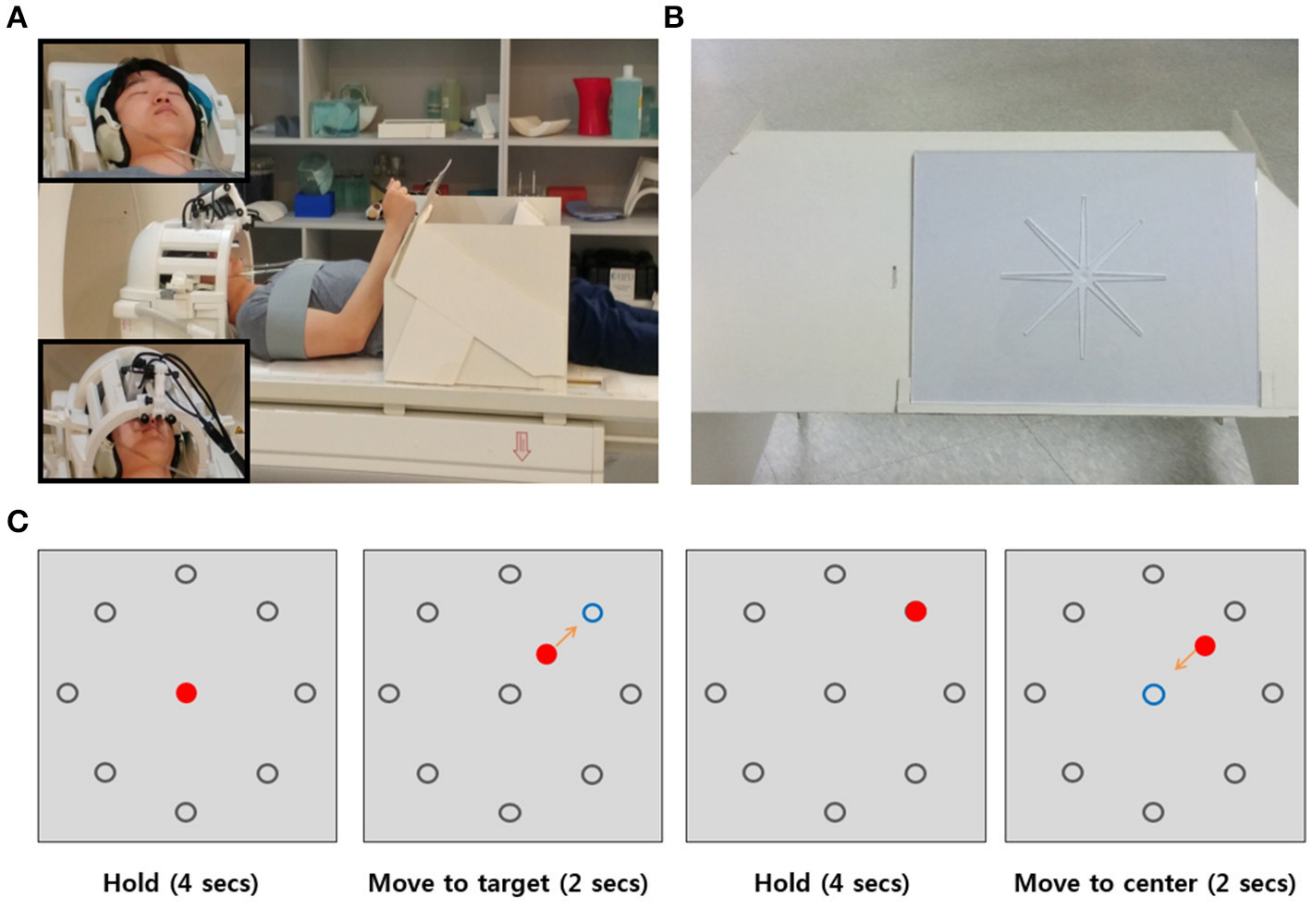
Setup and Experimental Design. **(A)** Participants laid in the scanner and put their arm on the arm-rest table to perform a center-out reaching task. To perform the task without moving their head, shoulder, or upper arm, the participant's head was stabilized with foam padding inside the head coil (upper left corner in **(A)** and fixed using cushioned head stabilizers placed on each side of the head coil (bottom left corner in **(A)**. As additional precautions against head movements and shoulder, the participant's shoulder was stabilized with a strap wrapping across the chest and shoulder. **(B)** The acrylic panel for the reaching movement. The carved route served to maintain a constant reaching direction for each trial. **(C)** Example sequence of a trial. There were eight gray target circles in the periphery of the center. The blue circle indicated the target position which to move and the red circle indicated the movement trajectory the participant used to move toward the target direction.

Each subject performed a center-out movement task. This task involved a total of six runs. In the task, subjects were instructed to move their right arm from a center position to one of eight target positions along a route carved into an acrylic panel (Figures 1A,B). The reaching movement was performed five times in each run, along with five no movement trials on the center position. Each run involved 40 trials for the reaching movements and 5 trials for the no-movement cases. No movement trials were pseudorandomly interleaved between reaching movement trials. The reaching movements in the experiment accounted for 240 trials in total (30 trials per each direction). Each trial lasted 12 s. One run lasted 9 min and 24 s.

In each trial, we showed the participants a gray circle in the center and eight gray circles on the periphery of the center circle on a screen. Initially, they were instructed to hold their arm at the center position which was the initial position before reaching their arm to the target position. After 4 s, one of the eight gray targets turned blue, which indicated the target direction to which to move. The participants had to move their arm toward the blue target position for 2 s. After reaching the target position, they were instructed to keep their arms still at the target position for 4 s. When the circle in the center turned blue, they moved their arm back to the initial center position (Figure 1C). The visual task was programmed with MATLAB Psychtoolbox-3 for windows (Brainard, 1997). The visual cues were presented with MR-compatible video goggles (Nordic Neuro Lab, Norway).

Before performing a center-out movement task, each participant was sufficiently trained to become familiar with the reaching movement inside the scanner and instructed to move smoothly and consistently. To perform reaching movements on the acrylic panel without moving their head, shoulder, or upper arm, the subject's head was stabilized with foam paddings inside the head coil and cushioned head stabilizers placed on each side of the head coil to reduce head motion. The subject's upper arms and shoulder were stabilized with a strap wrapping across the chest and shoulder (Figure 1A).

### Data preprocessing

Data preprocessing was performed using custom software written in Matlab (The MathWorks, Inc., USA). The first four volumes of each run were discarded automatically during the scanning process. We performed three-dimensional motion correction using the first volume as a reference, and the T2 anatomical image was coregistered to the functional image data by SPM5 (http://www.fil.ion.ucl.ac.uk/spm). No spatial smoothing was applied. The left primary motor cortex (M1) ROI was individually defined for each participant by converting the left M1 ROI of the standard MNI brain (Maldjian et al., 2003) to that of an individual brain using the SPM5 deformation toolbox. Voxels with extremely low signal intensity levels were removed. The fMRI signals were linearly detrended and passed through a high-pass filter using a cutoff frequency of 0.01 Hz within each run to remove low-frequency drift. We regressed out residual motion effects from the fMRI signals using six motion parameters (three translational parameters and three rotational parameters). The parameters were estimated using rigid body transformation between each functional image and a reference image during motion correction procedure by SPM. Signal intensities were normalized by removing the baseline from the fMRI signal of each voxel within each run and were averaged within each reaching movement trial after shifting the data by 3–5 s to compensate for hemodynamic delays in each case. We selected relevant voxels within the left M1 using sparse multinomial logistic regression (SMLR)-based feature selection. In a typical fMRI experiment, there are too many voxels in the brain compared to the number of samples that can be obtained. Too many voxels or features can lead to poor generalization performance by overfitting the learning model if all voxels are used as input features. Support vector machine (SVM) we used in this study for classification can avoid this problem by maximizing the margin and minimizing the classification error. However, the generalization performance of SVM is also decreased if too many irrelevant features are used to train the model. Therefore, we used one of voxel selection methods that can be used as a stand-alone tool box for voxel selection to improve the model performance by removing the irrelevant voxels. SMLR-based voxel selection was based on the classification performance and selection frequency as selection counting value (SC-value). Irrelevant voxels which have 0 SC-value obtained by SMLR-based feature selection were removed. SMLR-based feature selection was implemented by SLR toolbox (Miyawaki et al., 2008; Yamashita et al., 2008).

### Classification using multivoxel pattern analysis

We investigated whether human M1 voxels show directional sensitivity and whether the spatial patterns of the fMRI responses in M1 evoked by directional movements could be discriminable using MVPA based on linear classifier (Norman et al., 2006). The classification was performed with a linear support vector machine (SVM) classifier, one of the most popular classifiers in MVPA literature. The SVM was implemented by the LIBSVM toolbox (Chang and Lin, 2011) and we applied all of the default parameters of linear SVM (C = 1). The fMRI responses of voxels in left M1 ROI evoked by reaching movements were used for the classification. We evaluated the classification performance of the SVM using a leave-one-run-out cross-validation procedure. In each cross validation, one run (40 trials, 5 trials in each movement direction) was retained as a test dataset while the remaining five runs (200 trials, 25 trials in each movement direction) were used to remove the irrelevant voxels using the SMLR-based feature selection and to train the SVM classifier using the responses of selected voxels. This procedure was repeated 6 times until all runs were used as a test dataset. The classification performance was obtained by averaging the prediction accuracies across runs.

### Classification using encoding model

**Figure 2:**
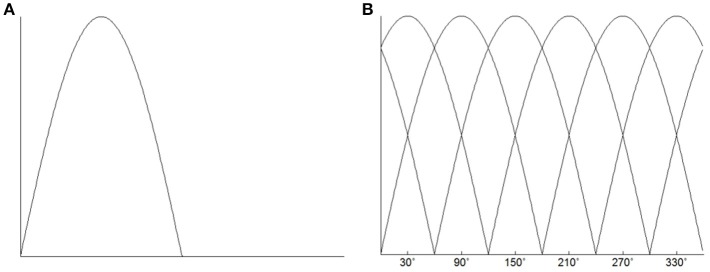
**(A)** Directional tuning curve. The basis tuning shape was modeled as a half-wave rectified sinusoidal curve. **(B)** Six directional tuning curves were used to characterize the response of each voxel. The response of the voxel could be fitted by a linear combination of the six half-wave rectified sinusoidal curves.

To characterize the responses of each voxel, we used a simple linear encoding model (Brouwer and Heeger, 2009) that has been used for color decoding in visual cortical areas. Recent evidence has showed that neurons in human M1 are directionally tuned (Hochberg et al., 2006, 2012; Truccolo et al., 2008; Collinger et al., 2013). The fMRI responses in human M1 were also found to be sensitive to movement directions (Eisenberg et al., 2010; Fabbri et al., 2010). Therefore, we could characterize the directional tuning of the response of each voxel. To characterize the directional tuning of each voxel, we assumed that the neurons in human M1 were directionally tuned, and the activity shape of the directionally tuned neurons was modeled as a half-wave rectified sinusoidal curve. The negative values of the tuning curve were set to 0 (Figure 2A). Given that there are a large number of neurons in each voxel, we also assumed a relationship between the fMRI responses and the neuronal activity. Previous studies provides evidence that fMRI responses are linearly related to the sum of the activity of all neurons within a voxel (Heeger et al., 2000; Rees et al., 2000). Although it was an oversimplification, we assumed a linear relationship between the fMRI response and the local neuronal activity. Therefore, the response of a voxel was the result of summing up responses of all neurons distributed in that voxel. The tuning function of the voxel then characterized as a linear combination of six half-wave rectified tuning curves. The six directional tuning curves is shown in Figure 2B. The tuning curve served as a basis function of the linear encoding model. The directional movement stimulus was an angular variable ranging from 0 to 2π.

The classification performance of the encoding model was also evaluated using a leave-one-run-out cross-validation procedure. In each cross validation, five of the six runs (*R*_1_, 200 trials, 25 trials in each movement direction) were used to remove the irrelevant voxels and to fit the encoding model using the responses of selected voxels, while the remaining one run (*R*_2_, 40 trials, 5 trials in each movement direction) was used to predict the movement directions using the fitted encoding model. The selected voxel responses *R*_1_ could be expressed as the weighted sum of six basis functions. The linear encoding model was given by

$$\begin{array}{l} R_{1}=Sw, \end{array}$$

where *R*_1_(*m* × *n*) is the measured voxel response matrix, *S* (*m* × *k*) is a response matrix of the six basis functions related to the reaching movement stimulus, and *w* (*k* × *n*) is a linear weight matrix. Let *m* denote the number of reaching movement trials, *n* denote the number of voxels, and *k* denote the number of the basis functions.

The weight matrix *w* was obtained using a regularized linear regression procedure to find an optimal weight to fit the encoding model more robust. The optimal weight matrix was given by

$$\begin{array}{l} ŵ=\;{\left(S^{T}S+\;\lambda I_{k}\right)}^{-1}S^{T}R_{1}, \end{array}$$

where *I*_*k*_ is the k-dimensional identity matrix and λ is the regularization parameter used. The lowest Bayesian information criterion (BIC) (Schwarz, 1978) was used to find the optimal value of the regularization parameter λ through a bootstrap method (Efron and Tibshirani, 1994). The parameter λ was optimized by using a regression function in RBF networks toolbox (Orr, 1996a,b).

The model was fit to the voxels individually using the linear combination of a set of basis functions. Therefore, we could estimate the response of each voxel and the spatially distributed pattern of the response across voxels activated by each movement direction. The reaching movement direction for which the predicted spatial pattern of voxel responses could be decoded by matching the most similar one with the observed spatial patterns of voxel responses. However, since there exists substantial variation in the measured voxel responses due to noise, we used the estimated response of the basis functions to match with the responses of the basis functions associated with a movement stimulus. The responses of basis function Ŝ were predicted using the estimated weight ŵ and test data *R*_2_ as follows:

$$\begin{array}{l} Ŝ=\;R_{2}{ŵ}^{T}{\left(ŵ{ŵ}^{T}\right)}^{-1} \end{array}$$

Because ŵŵ^*T*^ was close to singular in some cases, the inverse of ŵŵ^*T*^ was unstable. Therefore, we used regularization to estimate the inverse. The movement direction could then be predicted by comparing the estimated response patterns of basis functions Ŝ with known responses of the basis functions evoked by each of eight directional movements, and selecting the most similar patterns through an assessment of Pearson's *r*-values. Cross-validation step was repeated 6 times for all of the runs. The results based on the encoding model show the averaged classification accuracies across runs.

### Reconstruction using encoding model

MVPA and the encoding model were used to identify the movement directions. Furthermore, using the encoding model, the reconstruction of the movement direction was also performed by creating response of the basis functions for all possible movement directions from 0 to 360°. The reconstructed direction was estimated by matching the estimated response pattern Ŝ with the most similar one from the created response pattern. The reconstruction was also tested using a leave-one-run-out procedure. Five of the six runs (200 trials, 25 trials in each direction of movement) were used to remove the irrelevant voxels and to fit the encoding model using the responses of selected voxels. The remaining run (40 trials, 5 trials in each direction of movement) was used to test the reconstruction performance.

We further tested the reconstruction performance in the case when movement directions were not used to fit the encoding model in order to determine the feasibility of reconstruction for unknown movement directions. To this end, the reconstruction was tested using a leave-one-direction-out procedure. One of the eight directional movements (30 trials) was remained to test the reconstruction performance as the unknown direction. The other seven directions (210 trials, 30 trials in each direction of movement) were used to select the relevant voxels using the SMLR-based feature selection and then to fit the encoding model using the responses of selected voxels. This procedure was repeated 8 times to test the reconstruction of all of eight directional movements.

The reconstructed directions for each directional movement trials were spread out from the actual movement direction. Therefore, we used the angular variance (AV) to quantify the measurement of the angular dispersion of reconstructed directions. The AV was defined as AV = 1− ∥ *r* ∥, where ∥ *r* ∥ is the length of the mean angular direction which is obtained by means of vector addition of reconstructed directions. The quantity of AV lies in the interval [0, 1]. It is indicative of the spread in reconstructed directions. If the reconstructed directions were spread out evenly around all directions, the AV would be close to maximal, otherwise the reconstructed directions were concentrated completely in the actual movement direction.

To compare the reconstruction performance capabilities in the case when movement directions were used to fit the encoding model with when they were not used, we investigated the association between the results of the two reconstructed directions by computing the circular correlation coefficient ρ (Jammalamadaka and Sengupta, 2001), as follows,

$$\begin{array}{l} \rho =\;\frac{\sum_{i}\text{sin}\left(\alpha_{i}-\overset{-}{\alpha }\right)\text{sin}\left(\beta_{i}-\overset{-}{\beta }\right)}{\sqrt{\sum_{i}\text{si}{\text{n}}^{2}\left(\alpha_{i}-\overset{-}{\alpha }\right)\text{si}{\text{n}}^{2}\left(\beta_{i}-\overset{-}{\beta }\right)}} \end{array}$$

In this equation, α and β denote the reconstructed directions, $\overset{-}{\alpha }$ and $\overset{-}{\beta }$ denote the mean angular directions, and *i* denotes the number of reconstructed directions for each target direction. The correlations were obtained separately for each direction and by combining all directions for each subject.

To evaluate the reconstruction performance how they are reconstructed correctly to the actual movement directions, we computed the mean absolute error (MAE) between the reconstructed and actual movement directions across all of the reconstructed directions for individual subject. This is also computed in both cased when movement directions was included in fitting the encoding model and when they were used as unknown movement directions to compare the reconstruction performances. In this study, the reconstruction was performed based on single-trial directional movement. To investigate performance improvements when the directional movement was executed repeatedly, we computed MAE values of the mean angular directions obtained by averaging all reconstructed directions and compared the performances between the reconstruction based on single-trial and the trial-averaged reconstruction.

### Head motion effects

In fMRI experiments, head motion associated with motor tasks is a prominent source of noise which leads to fMRI data artifact, false detections, and misinterpretations of brain signals (Friston et al., 1996; Thesen et al., 2000; Yang et al., 2005; Culham et al., 2006). In this study, participants performed reaching-out movement task from a center to one of eight target directions. These repeated arm movements could have an effect on head motion sufficiently by generating constant movement patterns. Therefore, we investigated how head motion induced by participant-active reaching movements has an influence on the classification of directional movements. We used left M1 as a region of interest (ROI) because all reaching movements by right-handed participants were performed with the right hand. Movements performed with the dominant hand was associated with a greater activation compared with those of the non-dominant hand in the contralateral motor cortex. (Dassonville et al., 1997; Fabbri et al., 2010; Grabowska et al., 2012). Moreover, although highest directional selectivity in the right parietal reach region for both left and right hand movements was observed, executed movements performed with the right hand as well as observed and imagined movements led to non-significant activations in the ipsilateral motor cortex (Dassonville et al., 1997; Fabbri et al., 2010). The movement activation in the right M1 would have been trivial even though it reflects any neural motion related signal by hemispheric interactions. The activation in ipsilateral M1 uninvolved in the task would reflect the head motion effects dominantly rather than hemispheric interactions. Therefore, we chose the right M1 as a control region to identify the head motion effect by the reaching movement. The performance of the control region would yield some evidence for residual head motion. The classification using MVPA and the encoding model was conducted from the right M1. The procedures were exactly identical, except for the spatial prior mask from the MNI atlas left and right M1.

## Results

### Classification performance

**Figure 3:**
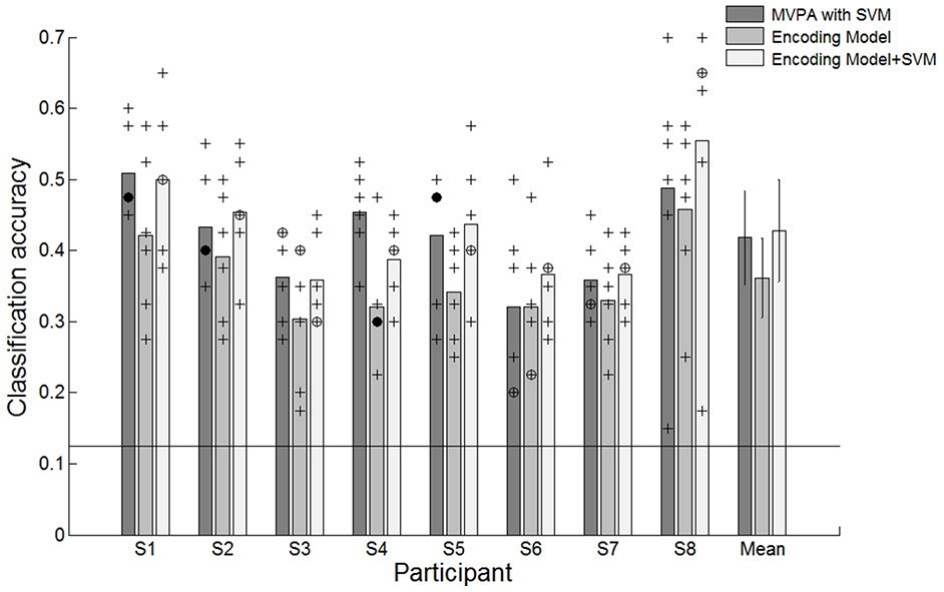
Classification accuracies for individual participants and average accuracies across all participants using the conventional decoding approach based on the linear SVM classifier, the linear encoding model and combination of the encoding model and the SVM classifier. ^+^Indicated the performance of each run which was used test dataset during cross validation. ^⊕^Indicates the performance of each run was overlapped twice. ^•^Indicates the performance of each run was overlapped three times. The solid line indicates the chance level of 12.5%. The error bars in averaged accuracies (mean) indicate the SDs across participants.

We evaluated the classification performance of MVPA based on SVM classifier and the encoding model via a leave-one-out cross-validation scheme. We excluded one run (40 trials) from the set of six runs to test the performance and trained the SVM classifier or the encoding model using the remaining five runs (200 trials). The classification result indicated that reaching movements toward eight different directions could be decoded from the spatially distributed patterns of the voxel responses. The classification performances of both MVPA and the encoding model were significantly greater than the chance level of 12.5% in all participants (Figure 3). Each point indicates the classification performance of each run which was used as test dataset during cross validation. Average accuracies across all participants for the MVPA and the encoding model were 41.8 and 36.1%, respectively. In comparison, MVPA outperformed the encoding model. These results indicated that decoding approaches using the linear classifier outperform the encoding model when used to classify brain states evoked by certain executed movements. However, such a classification-based technique shows limitations when used for decoding complex motor actions. It is impractical to measure brain activity given many states which are possible. Compared to classification-based technique, the encoding model is more applicable to decode the complex motor actions by being able to predict unknown brain states as well as to identify known states.

In the encoding model, the classification performance was less than that of MVPA. Reaching directions were predicted by matching the estimated response pattern Ŝ with the highest correlated one among response patterns by each of eight directional movements through an assessment of Pearson's *r*-values. The decision boundary used to classify the reaching movement directions was not optimized in the encoding model. Therefore, we further investigated the performance validity of the encoding model by combining the encoding model with the SVM classifier. To use the SVM classifier in the encoding model, we trained the SVM classifier using the estimated response pattern of basis functions from the encoding model that was fitted by training data and then tested the classification performance capabilities. The average performance across all participants improved from 36.1 to 42.8% (Figure 3). There were no statistically significant performance differences between MVPA and the combined model. These results indicated that the encoding model could be used to decode which direction had been moved, and these performances confirmed the validity of the encoding model for classification.

### Reconstruction performance

**Figure 4:**
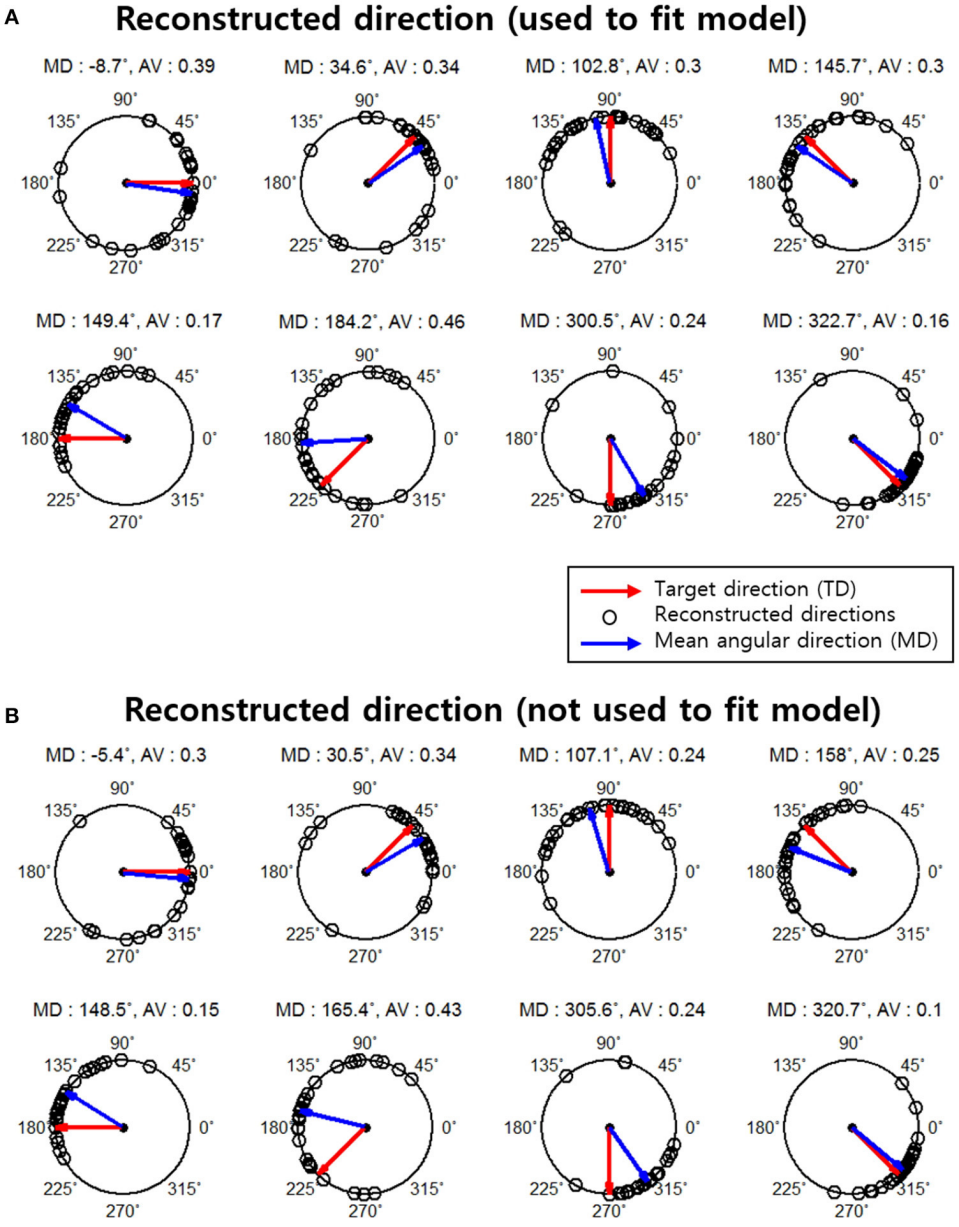
Reconstruction results for subject 1. The red arrows indicate the actual movement directions (target directions). Black points indicate the reconstructed directions on a single-trial basis (total 30 reconstructed directions in each movement direction. The blue arrows represent the mean angular direction for all reconstructed directions in each target direction. The mean angular direction was obtained by means of vector addition. The angular variance (AV) was defined as AV = 1− ∥ *r* ∥ and the interval of the AV was [0, 1]. ∥ *r* ∥Indicates the length of the mean angular direction. **(A)** Results of the reconstructed directions that were used to fit the encoding model. **(B)** Results of reconstructed direction that were not used to fit the encoding model.

The result of reconstructed direction for the first subject (S1) is shown in Figure 4A. The red arrows represent each actual movement direction to which the subject moved. Each black point indicates all reconstructed directions across all runs (30 trials per each movement direction). The blue arrows represent the mean angular direction for all reconstructed direction trials. The results indicated that most of the reconstructed directions were clustered near each actual movement direction and that some reconstructed directions had large errors.

**Figure 5:**
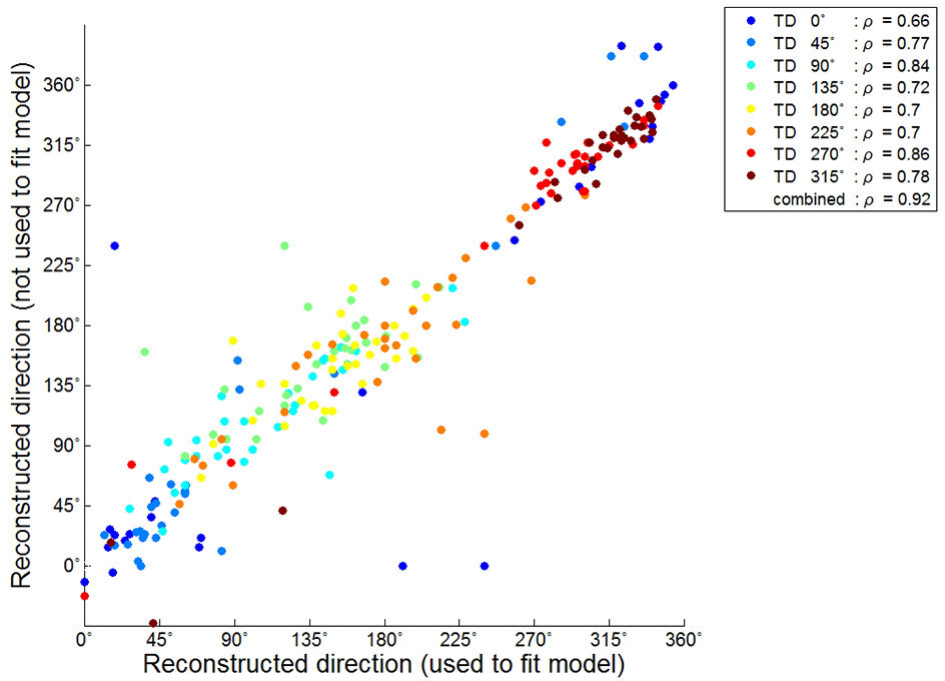
Circular correlation result between the reconstructed directions used to fit the model and those not used for subject 1. Each color point corresponds to the reconstructed directions in each target direction. ρ Indicated the circular correlation coefficient obtained separately for each target direction and that after combining over all directions in each subject.

We further investigated the reconstruction capabilities of when unknown directions are used. This reconstruction result is shown in Figure 4B. The results of the reconstructed direction indicated patterns similar to those of the reconstructed directions used to train the encoding model (Figure 4A). Even so some reconstructed directions had large errors showed similar patterns. To quantify this, we investigated the association between the results of the two reconstructed directions using the circular correlation coefficient ρ. The correlation result for the first subject (S1) is illustrated in Figure 5. For this subject, the reconstructed directions of when movement directions are used to fit the model were highly correlated with the reconstructed directions of when the movement directions are not used to fit the model for each target direction. The distributed pattern of the combined reconstructed directions over all directions also showed a highly significant correlation of ρ = 0.92. This result confirmed that the reconstructed directions by the encoding model revealed a similar pattern regardless of whether or not the reaching directions were included when fitting the model. The correlation results for all subjects are shown in Table 1.

**Table 1:** Circular correlation coefficients.

**Target direction**	**S1**	**S2**	**S3**	**S4**	**S5**	**S6**	**S7**	**S8**
0°	0.66	0.33	0.66	0.19	0.86	0.32	0.5	0.66
45°	0.77	0.77	0.53	0.47	0.5	0.31	0.35	0.82
90°	0.84	0.77	0.77	0.53	0.91	0.55	0.53	0.46
135°	0.72	0.8	0.75	0.2	0.71	0.79	0.49	0.7
180°	0.7	0.79	0.77	0.07	0.81	0.56	0.17	0.68
225°	0.7	0.46	0.68	0.54	0.15	0.23	0.63	0.29
270°	0.86	0.82	0.77	0.75	0.76	0.75	0.45	0.44
315°	0.78	0.67	0.89	0.29	0.72	0.78	0.51	0.52
Combined	0.92	0.9	0.7	0.58	0.8	0.7	0.81	0.71

**Figure 6:**
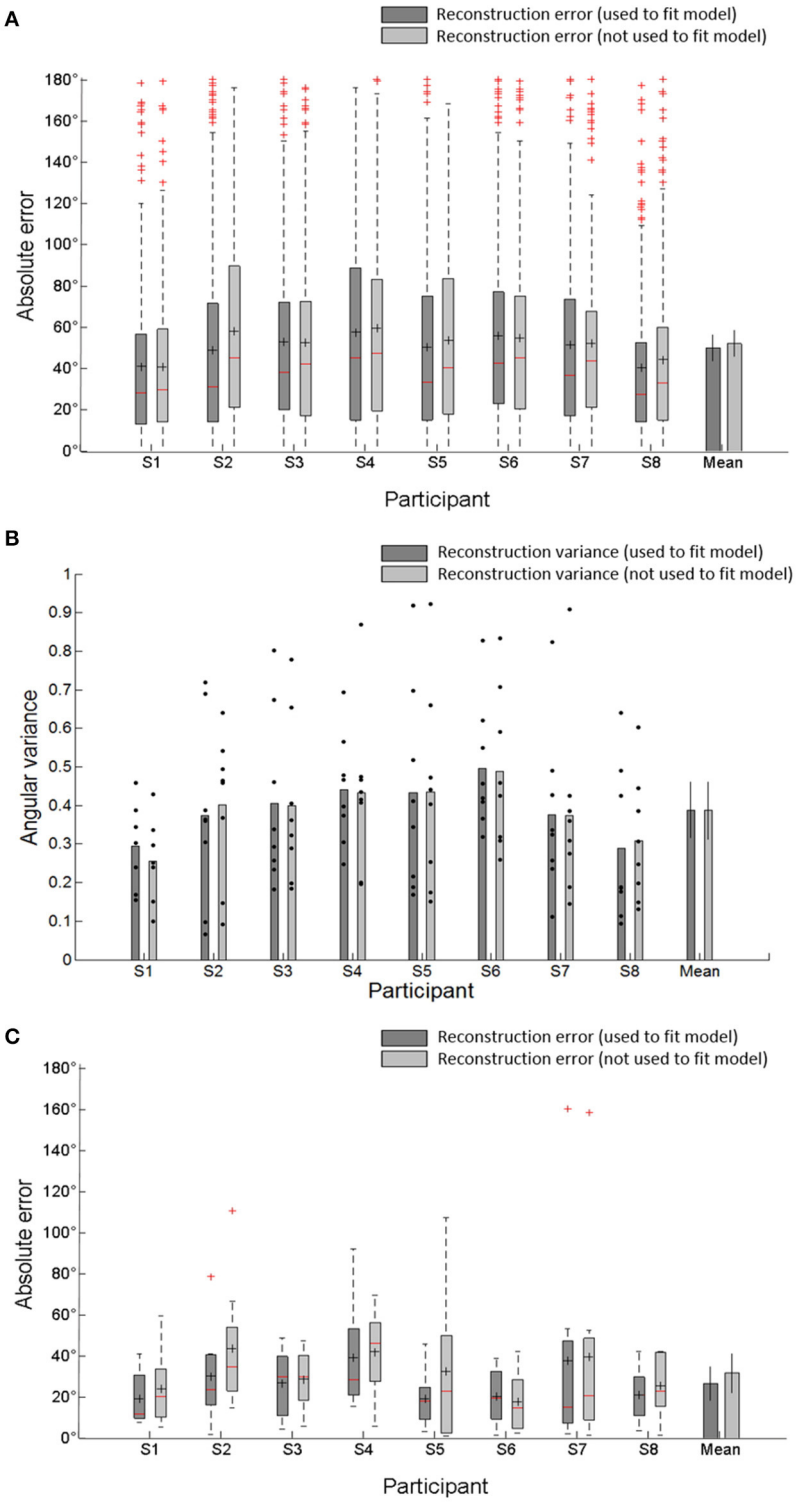
**(A)** Absolute error between the reconstructed directions and the target directions by combining all reconstructed directions in each subject. It represents the distribution of reconstructed direction errors in each subject. The central reds line in central boxes indicate the median of the reconstruction errors. Black + in the central box indicate the mean of reconstruction's absolute errors (MAE). The central box represents the central 50% of the absolute errors. Its lower and upper boundary lines are at the 25%/75% quantile of the errors. Vertical lines from the central box indicate the remaining data outside the central box except outliers (red +). Bar plot indicates average MAE and SD across all participants. **(B)** Angular variance (AV) of reconstructed directions. Black dots indicated angular variance of each target directions which was used to reconstruct movement directions. The error bars in averaged variance (mean) indicate the SDs across participants. **(C)** Absolute error between mean angular directions obtained by averaging all reconstructed directions and target directions in each subject and the average MAE and SD across all participants.

To evaluate the reconstruction performance capabilities of when unknown directions are used and not used, we computed the absolute error and mean absolute error (MAE) between the reconstructed and actual movement directions (target directions) across all of the reconstructed directions for individual subject and the MAE across all subjects. When unknown directions were used as test directions for the reconstruction, the errors were larger than when known directions were used (Figure 6A). That is, the reconstruction accuracy when unknown directions were used was less than the accuracy when known directions were used. However, there were no significantly different reconstruction errors between both cases. The shape of skewed distribution toward target directions also showed that the reconstructed directions were clustered near the target directions. The angular dispersion of reconstructed directions when unknown movement directions were used as the test directions was also similar to the dispersion of reconstructed directions when known directions were used (Figure 6B). These reconstruction results confirmed the validity of the encoding model to reconstruct unknown movement directions. Furthermore, to compare the performance between the reconstruction based on each trials and the trial-averaged reconstruction, we also computed the MAE values of the mean angular direction obtained by averaging all reconstructed trials. The trial-averaged reconstructed performances were much higher than the performances based on the single trials (Figure 6C). The average MAE of the mean directions across all participants reduced from 49.8 to 26.6° when known directions were used to test the reconstruction and from 51.9 to 31.6° when unknown directions were used. This indicated that the trial-averaged reconstruction approach could more efficiently improve the reconstruction performance than the single-trial approach.

### Head motion effects

**Figure 7:**
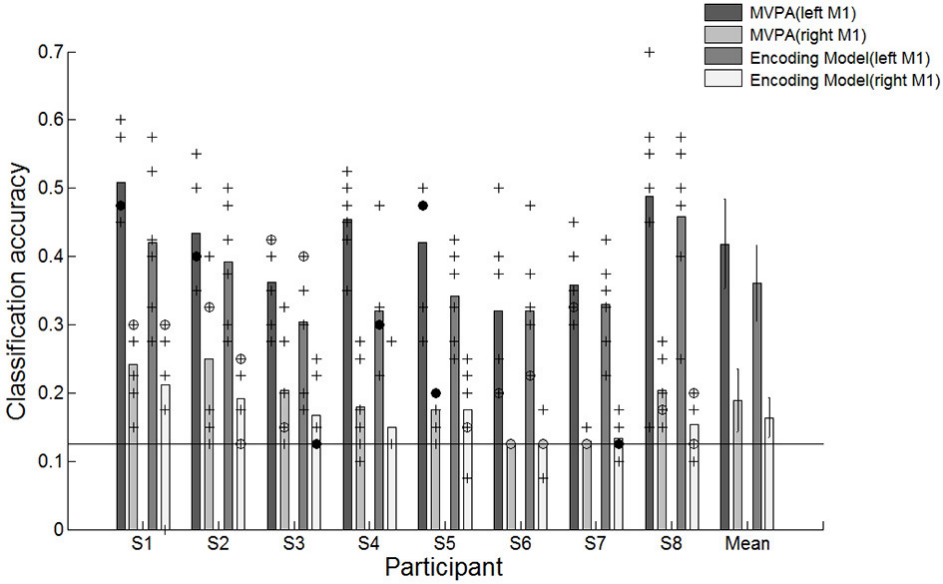
Classification accuracies for individual participants and average accuracies across all participants using different motor cortex regions (left and right M1). ^+^Indicates the performance of each run which was used test dataset during cross validation. ^⊕^Indicates the performance of each run was overlapped twice. ^•^Indicates the performance of each run was overlapped three times. 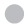
Indicates the performance of each run was overlapped over four times. The solid line indicates the chance level of 12.5%. The error bars in averaged accuracies (mean) indicate the SDs across participants.

We evaluated the classification performance from the right M1 ROI via a leave-one-out cross-validation to identify head motion effects by the reaching movement task. One run (40 trials, 5 trials in each movement direction) from the set of six runs was excluded to test the performance. Remaining runs were used to train the SVM classifier and encoding model (200 trials, 25 trials in each movement direction). It was repeated 6 times until all runs were used to test the performance. The classification performance compared the left M1 and the right M1 is shown in Figure 7. The average accuracies across all participants for the left and right M1 based on MVPA were 41.8 and 18.9%, respectively, and the average accuracies for the left and right M1 based on encoding model were 36.1 and 16.4%, respectively. The small discriminability in right M1 could have been influenced by a residual motion signal that was not captured by the rigid body transformation for motion correction. Because each fMRI volume is slice-wise assembled over time, the rigid body transformation may not properly estimate the actual head movements between and within slice acquisition. Compared to average performances of the right M1 across participants, the left M1 is significantly higher than the right M1 for both MVPA and encoding model. The results indicated that the directional movements measured on the right M1 were insufficient for discrimination. Therefore, the effect of head motion does not have a significant effect on the classification and reconstruction performance.

## Discussion

It is well known that motor cortical neurons encode various movement features, such as directions of movement (Georgopoulos et al., 1982), hand positions (Georgopoulos et al., 1984), velocities (Moran and Schwartz, 1999), and force (Taira et al., 1996; Sergio and Kalaska, 1998) to generate a variety of complex motor actions. The directional tuning of motor cortical neurons is one of the most important factors related to reaching movements. In this study, we investigated whether an approach using the directional tuning could be applied to fMRI responses to reconstruct movement directions non-invasively. The responses of voxels reflect pooled activity of neuronal populations because a large number of neurons with different movement selectivities are distributed within each fMRI voxel. Thus, the directional sensitivity of the overall response would be weaker than that of single motor neurons by averaging out the sensitivity and adding the noise. Nevertheless, the pooled response of the neurons consistently characterized with directional sensitivity due to the spatial distribution of the neurons not being uniform. This generates distinct spatial patterns of responses across multiple voxels for the reaching movements and makes it possible to decode the directional movements from the distributed response patterns. Earlier fMRI work was also revealed that individual voxels in human M1 have directional tuning properties by computing the coefficient of variation of five directions of reaching movements for each voxel (Eisenberg et al., 2010). In this study, we verified that when using decoding approaches based on a linear classifier, reaching movements toward eight directions were distinguishable with high accuracy from the spatially distributed patterns of responses across an array of voxels measured in human M1. Although fMRI signals were influenced by head motion effect, this indicated that responses of M1 voxel were directionally sensitive for movement directions. Therefore, we assumed that directional tuning was encoded in M1 voxel. The responses of each voxel could be simply modeled using the directional tuning property of the motor cortical neurons. The directional tuning in each voxel was estimated by a linear combination of the six sinusoidal curves. Thus, directional movements could also be predicted using the estimated responses of M1 voxels.

As a result of the classification of reaching movement directions, the encoding model also demonstrated high performance capabilities for all participants which were also comparable to MVPA. Although the classification performance of MVPA was better than that of the encoding model, the encoding model is more applicable to predict complex motor information. The classification-based decoding approaches are used to classify brain activity into a specific experimental stimuli or tasks, while the encoding model could predict brain activity without any prior stimulus. To this end, we performed the reconstruction of unknown directional movements and compared the reconstruction results of the encoding model which was fitted by seven directional movements with those of the encoding model which was fitted by all eight directional movements. The reconstruction results demonstrated that they were clustered around the target direction to which the subject moved. Furthermore, the distributed patterns of reconstructed directions showed a highly significant correlation regardless of whether the encoding model was estimated by seven or eight movement directions. This indicated the potential feasibility of decoding any possible directions over the eight movement directions given during experiment task.

Recent fMRI studies have advanced beyond the classification of cognitive states from experimentally predefined stimulus. However, such advanced fMRI studies have mostly been conducted using visual stimuli such as visual images (Kay et al., 2008; Miyawaki et al., 2008; Naselaris et al., 2009) and dynamic natural movies (Nishimoto et al., 2011). Thus, we applied such decoding methods to predict motor information about directional motor movements in human motor cortex. In the present study, we used an intuitive and simple encoding model to classify and reconstruct the movement directions. The encoding model was defined based on directional tuning properties in human motor cortex to estimate fMRI responses in each voxel evoked by a center-out reaching task. We could perform the identification and the reconstruction of movement directions using the encoding model. During the reaching task, head motions by repeated reaching movements could have an influence on the identification performance. However, the left M1 showed significantly higher performance than the right M1, and the performance from the right M1 was closed to chance level. This implies that motor information associated with directional motor movements is encoded in the responses of voxels, and fMRI responses in human M1 are directionally selective. Therefore, this result suggests that decoding approaches based on the encoding model could be applied to use motor information. Nonetheless, precise and detailed encoding models for decoding complex motor actions in real life can be considered to be used more practically. In the present study, the identification and reconstruction were performed based on single-trial basis. Reconstructed directions based on single-trial were spread out around target directions. It could cause a lot of errors to use motor information practically. Thus, using the mean angular direction predicted by averaging movement trials could reduce the reconstruction error much more than using reconstructed directions on the single-trial basis. To improve the decoding accuracy even more, a trial-averaged procedure in which movement executions are repeatedly performed is not suitable. Therefore, in the place of movement executions, decoding approaches based on motor imagery could be considered as future work. Furthermore, a future investigation with the encoding model would need to consider a variety of features related to complex movements such as directions, velocities, positions, and force.

## Author contributions

SN performed a design of the fMRI experiment, the acquisition of the data, analysis, and interpretation of data for the work. He wrote manuscript. DK performed a conception of the work, editing draft of manuscript, final approval of the version to be submitted.

### Conflict of interest statement

The authors declare that the research was conducted in the absence of any commercial or financial relationships that could be construed as a potential conflict of interest.
